# Downregulation of Sprouty homolog 2 by microRNA-21 inhibits proliferation, metastasis and invasion, however promotes the apoptosis of multiple myeloma cells

**DOI:** 10.3892/mmr.2015.3567

**Published:** 2015-03-30

**Authors:** JIN-HANG WANG, WEN-WEN ZHOU, SHI-TONG CHENG, BO-XIN LIU, FU-RONG LIU, JIAN-QING SONG

**Affiliations:** 1 Department of Laboratory Medicine, The First Hospital Affiliated to China Medical University Clinical Laboratory, Shenyang, Liaoning 110000, P.R. China; 2 Department of Cell Biology, China Medical University, Key Laboratory of Medical Cell Biology, Ministry of Public Health, Shenyang, Liaoning 110000, P.R. China

**Keywords:** multiple myeloma, microRNA-21, sprouty homolog 2, proliferation, invasion

## Abstract

The aim of the present study was to assess the effects of sprouty homolog 2 (SPRY2) gene regulation by miR-21 on the occurrence, development and tumor metastasis in multiple myeloma (MM). The miR-21 expression lentiviral vector (LV)-anti-miR-21 and a liposome transfection method were used to screen MM cell lines with stable silent SPRY2. Real-time quantitative polymerase chain reaction (PCR) and western blot analyses were used to detect SPRY2 expression and miR-21 protein expression levels. An MTT assay was used to assess cell proliferation. Flow cytometry was used for analysis of cell cycle. A scratch test/wound healing assay was used to detect the cell migration ability. A Transwell assay was used to detect the cell invasion ability. Real-time quantitative PCR and western blot analysis showed that in the MM cell lines with high endogenous miR-21 expression (RPMI8226 and KM3), SPRY2 expression was significantly lower. Conversely, in the U266 cell line with low endogenous miR-21 expression, SPRY2 expression was significantly higher, and the gray values of miR-21 and SPRY2 protein in the respective cell lines showed statistically significant differences (P<0.01). Following transfection of U266 cells, the expression of miR-21 in the U266/LV-anti-miR21 lentiviral multiplicity of infection (MOI) 20 group and -MOI 40 group decreased significantly compared with that in the untransfected U266 group (P<0.05). SPRY2 protein expression in U266 cells transfected with miR-21 mimics was significantly reduced compared with that in the non-transfected (untreated) group and the negative control-transfected group (P<0.01). An MTT assay showed that compared with the non-transfected and negative control groups, the cell growth rate as well as the proliferation rate were significantly decreased in the transfection group 48, 72 and 96 h after transfection (P<0.01). Flow cytometric analysis showed that 48 and 72 h after transfection of U266 cells with miR-21 mimics, the apoptotic rates were (24.7±1.97 and 38.6±1.56%) in the U266 group, (27.3±1.72 and 37.3±1.59%) in the siRNA group and (12.7±1.27 and 22.1±1.63%) in the U266/miR-21 group. Compared with the two control groups, the apoptotic rate in the U266/miR-21 group was significantly decreased and the G_0_/G_1_ phase cell population was significantly reduced (P<0.05). Scratch experiments showed that the cell migration ability was significantly reduced in the transfection group 24 and 48 h after transfection (P<0.05). A Transwell invasion assay confirmed that the number of U266 cells which migrated through a Matrigel-covered polyphosphate membrane significantly decreased in the transfection group 24 and 48 h after transfection. The cell-penetrating ability was also significantly decreased (P<0.05). In conclusion, the downregulation of SPRY2 gene expression mediated by miR-21 promotes the proliferation and invasion of MM cells *in vitro*, suggesting that miR-21 may be a novel potential molecular therapeutic target in the treatment of MM.

## Introduction

Multiple myeloma (MM) is a type of malignant plasma cell and has a high incidence in malignant tumors of the blood system, accounting for ~2% of the total mortality of cancer (1). In China, the incidence of MM accounted for 10% of total hematopoietic system cancers with an upward trend year by year. Although treatment strategies changed from traditional chemotherapy and autologous hematopoietic stem cell transplantation to novel targeted drug therapy, the outcome was not improved (2). Therefore, the identification of mechanisms underlying the regulation of the malignant behavior of MM and key genes in disease progression has the greatest significance for the establishment of novel therapeutic strategies and improvement of the prognosis in patients. In-depth study of small non-coding RNA molecules confirmed that microRNA (miR) has an important regulatory role in cell proliferation, differentiation, metabolism, apoptosis and development processes (3). Murphy *et al* (4) found that miR-21 is closely associated with the tumor and is able to adjust SPRY2 expression. SPRY2 is a member of the signaling pathway-specific inhibition protein sprouty (SPRY) family. According to their differential sequences, SPRY proteins were divided into four subtypes (SPRY1, -2, -3 and -4). The SPRY2 protein contains 315 human amino acid residues (35 kDa), with the C-terminal residues 178–282 being rich in cysteine. Due to its significant biological effects (5–8), SPRY2 has become a research hotspot. The present study intended to establish MM cell lines with stably silenced SPRY2 using RNA interference technology. Under *in vitro* conditions, changes in the proliferation and invasion ability were detected in myeloma cells. To investigate the occurrence, development and transfer process of MM, a novel molecular targeted therapy was established to provide a reliable basis for research.

## Materials and methods

### Instruments and reagents

ABI7500 real-time polymerase chain reaction (PCR) instrument (Applied Biosystems Inc., Life Technologies, Thermo Fisher Scientific, Waltham, MA, USA). A NanoPhotometer nucleic acid and protein ultraviolet detector (NanoPhotometer^®^ Pearl; Implen GmbH, Munich, Germany) and a 3K18 type low temperature high speed centrifuge (Sigma, Osterode am Harz, Germany) were used. The UVP GelDoc-It 310 gel imaging analysis system was purchased from Shanghai Kunke Co., Ltd. (Shanghai, China). TRIzol reagent, LA Taq DNA polymerase and lipid Lipofectamine 2000 (Invitrogen Life Technologies, Carlsbad, CA, USA) were used. The miRNeasy Mini kit serum total RNA extraction kit was from QIAGEN Inc. (Hilden, Germany). For cell culture, 10% FBS RPMI 1640 medium and DMEM culture medium (Hyclone, GE Healthcare, Little Chalfont, UK) were used. Agarose gel extraction kit and mir-21qPCR primer kit were purchased from Takara Bio Inc. (Otsu, Japan). Lentiviral vector LV-anti-miR-21 and control vector were from Shanghai SBO Medical Biotechnology Co. (Shanghai, China). SPRY2 eukaryotic expression vector was purchased from Origene (Rockville, MD, USA) and microRNA-21 mimics and inhibitors were from Biomics Biotechnologies (Nantong) Co., Ltd. (Nantong, China).

### Construction of plasmids

Prior to construction of the miR-21 lentiviral expression vector LV-anti-miR-21, the miR-21 precursor pre-miR-21 sequence was obtained using the miRBase (http://www.mirbase.org) database. Primer synthesis was performed by Shanghai Jierui Bio-Engineering Co., Ltd. (Shanghai, China). The upstream primer was miR-21 forward, 5′-CCGGTTCAACATCAGTCTGATAAGCTATTTTTTG-3′, and the downstream primer was mir-21 reverse, 5′-AATTCAAAAAATAGCTTATCAG-3′. DNA containing the pre-amplified sequence was used as the template for PCR amplification and the target-resulting fragment was used for *Xho* I and *BamH* I (Promega Corp., Madison, WI, USA) double digestion. The fragment was connected with the lentiviral expression vector LV-anti plasmid, and the connection reaction conditions were as follows: 1 *µ*l digested product, 1 *µ*l LV-anti plasmid vector, 6 *µ*l nuclease-free water, 1 *µ*l 10x ligase buffer and 1 *µ*l T4 DNA ligase (Promega Corp.) for 22°C in a water bath overnight. The ligation products were transfected into competent DH5 cells, the monoclonal colonies were selected and inoculated, and they were placed in a 37°C-thermostat overnight. Plasmids were extracted using a plasmid extraction kit, and *Xho* I and *BamH* I were prepared for restriction enzyme digestion. The reaction conditions were as follows: 17 *µ*l DNA, 2 *µ*l 10xPCR buffer, 0.5 *µ*l *Hind* III and 0.5 *µ*l *BamH* I. The reaction was performed at 37°C for 3 h. The bacterial liquid was sent to Invitrogen Life Technologies for sequencing.

### Establishment of stably SPRY2-silenced MM cells

LV-anti-miR-21 expression vector and unloaded cytomegalo-virus (CMV)-green fluorescent protein (GPF)-LV were added to a 24-well culture plate containing U266 cells (500 *µ*l/well; 7.0×10^4^ cells/ml) for 8 h of infection. The viral supernatant was replaced with appropriate medium, and 24–48 h after infection, green fluorescence was observed under a fluorescence microscope (BX51; Olympus Corporation, Tokyo, Japan). The culture was expanded following infection and cells in the logarithmic growth phase were collected and subjected to Aldefluor screening (Biowish Co., Ltd., Jiangsu, China) for 24 h. Cells containing the target gene and the cells transfected with an empty vector were isolated and re-cultured. When the cells were in the logarithmic growth phase, they were divided into three groups, namely: The U266/un group (untreated), the U266/GFP group containing the unloaded virus CMV-GFP-LV, and the LV-anti-miR-21 group, which was infected with the target gene. Following 24 h, G418 (1 mg/ml; Cian Wolsen Biotechnology Co., Ltd., Shanxi, China) screening was performed for 24 h and the clones were further cultured for subsequent experiments.

### Cell culture, transfection and G418 screening

The human myeloma cell lines U266, KM3 and RPMI8226 were provided by the Department of Cell Biology of China Medical University (Shenyang, China) The cells were between passages 1:2 and 1:4. RPMI 1640 medium containing 10% FBS was used for the culture of the myeloma cell lines U266, KM3 and RPMI8226, and they were placed in a humidified incubator at 37°C and 5% CO_2_ for subculture. When cells reached 80% confluency, cells at the logarithmic growth phase were collected. Lipofectamine 2000 was used to mediate G418 transfection of U266 cells. The cells were added and cultured in complete medium. Cells in the logarithmic growth phase were seeded in 12-well plates at a density of 1×10^5^ cells, and were divided into four groups: miR-21 mimics group (transfected with miR-21 mimics), miR-21 inhibitor group (transfected with anti-miR-21), untreated group (untransfected cells) and small interfering (si)RNA negative control (NC) group. Three wells were set for each group. The transfection concentration was 100 nmol/l, and 6 h after transfection, the medium was replaced with normal medium. 24 h later, DMEM containing 10% FBS and G418 was added for selection, and clones were obtained after two weeks of screening, followed by culturing of the clones for the subsequent experiments.

### miR-21 expression detected by real-time quantitative (RT-q)PCR

The TRIzol method was used to extract the total length RNA from the cells. The methods were according to the miR-21 RT-qPCR method by Chen *et al* (9). Briefly, the process was as follows: According to instructions of QIAGEN’s miRNeasy Mini kit, 200 *µ*l serum was used to extract total RNA from the cells, and RNA was stored at −80°C. The reverse transcription kit (Promega Corp.) was used for reverse transcription. PCR was conducted using an ABI7500 Real-Time PCR Instrument (Applied Biosystems Life Technologies, Foster City, CA, USA). PCR conditions were as follows: 95°C for 10 min, 95°C for 15 sec, 55°C for 15 sec, 72°C for 20 sec, for 40 cycles. Each tube contained 20 *µ*l PCR reaction mixture. According to the comparative threshold method by Livak and Schmittgen (10), Ct values were read, and the expression quantity of the targeted miR-21 gene was expressed using the 2^−ΔΔCt^ formula, with ΔΔCt=experimental group (Ctt_arget gene_-Ct_housekeeping gene_) - control group (Ct_target gene_-Ct_housekeeping gene_). Each condition was repeated in three wells, and the experiment was repeated three times.

### Western blot analysis

The cells in the logarithmic growth phase and with 80% confluency were collected, and radio-immunoprecipitation assay lysis buffer containing protease inhibitors (Beyotime Institute of Biotechnology, Haimen, China) was used for conventional extraction of total cellular protein. A bicinchoninic acid protein assay kit (Beyotime Institute of Biotechnology) was used for protein quantification. 5x loading buffer was added prior to sampling at 100°C for 5 min of boiling, and following cooling, the samples were filled. 10% SDS-PAGE was performed, protein was then transferred to a nitrocellulose membrane (Beyotime Institute of Biotechnology), 5% skim milk was added, and membranes were agitated in a sealed container for 1 h. The primary antibodies were then added: Rabbit polyclonal SGOL1 (1:100; cat. no. R4392; Ucallm, Beijing Biocoen Biotechnology Co., Ltd., Beijing, China) and mouse monoclonal GAPDH (1:5,000; cat. no. A01020; Abbkine, Inc., Redlands, CA, USA), and the membranes were incubated in a sealed container with agitation at 4°C overnight. Phosphate-buffered saline (PBS) containing Tween 20 (PBST; Beyotime Institute of Biotechnology) was used for washing the gels three times for 10 min each. Horseradish peroxidase-labeled goat anti-mouse antibody (1:5,000; cat. no. Ov02-03-02) and horseradish peroxidase-labeled goat anti-rabbit antibody (1:2,000; cat. no. Ov03-03) (Luoyang Baitaike Biotechnology Co., Ltd., Luoyang, China) were added and gels were incubated at room temperature for 1 h. PBST was then used for washing three times, gels were developed using enhanced chemiluminescence and visualized using a Gel imager camera (Bio-Rad Laboratories, Hercules, CA, USA). The target protein ratio was calculated from the grayscale ratio (miR-21/GAPDH or SPRY2/GAPDH).

### Cell proliferation ability detected by the MTT method

Cells in the logarithmic growth phase were collected following transfection in each group. Single-cell suspensions were prepared, the cell density was adjusted to 3×10^4^/ml, and cells were inoculated in collagen-coated 96-well plates with each group set in three parallel wells. 0, 24, 48 or 72 h following inoculation, 20 *µ*l MTT solution (5 mg/ml; Beyotime Institute of Biotechnology) was added to each well, cells were cultured for 4 h, and the supernatant was discarded. Dimethylsulfoxide (200 *µ*l/well; Beyotime Institute of Biotechnology) was added and plates were agitated to dissolve the formazan crystals over 10 min. An ELISA plate reader was used to detect the absorbance (A) values at 570 nm wavelength, with a blank well set as zero. The cell growth curves were drawn, and the cell proliferation inhibition rate was calculated from the optical density (OD) as: Inhibition rate (%) = (1-OD_experimental g roup_/OD_control group_) ×100%.

### Flow cytometric analysis of the cell cycle

The cells in each group were collected at 24, 48 and 72 h following transfection, and cold PBS was used to wash cells three times. The cells were resuspended in 500 *µ*l pre-cooled binding buffer, and the concentration was adjusted to 5×10^6^ ml. 100 *µ*l of the cell suspension was added to flow cytometry tubes and 5 *µ*l Annexin V-fluorescein isothiocyanate (Beyotime Institute of Biotechnology) was added. Following mixing, samples were incubated at room temperature in the dark for 15 min, and 5 min prior to the measurements, 5 *µ*l 10 mg/l propidium iodide (PI) dye (Beyotime Institute of Biotechnology) was added. Flow cytometry (FACScan; BD Biosciences, Franklin Lakes, NJ, USA) was used to determine the cell cycle distribution. Each sample was repeated three times. CellQuest FCS 3.0 software (BD Biosciences) was used for data analysis.

### Detection of cell migration using wound healing assay

Following transfection, the cells were collected and seeded in 48-well plates (1×10^6^/well). When the cells merged into a monolayer, a sterile 200 *µ*l pipette tip was used to carefully cause a line-shaped scratch at the surface of the cell layer. PBS was used for washing twice. An inverted microscope (BX51; Olympus Corporation) was used to observe the non-suspended or free cells at the borders of the scratches. The degree of scratch healing was observed and images were captured in each group (reflecting cell migration) at 0, 24 and 48 h. The cell migration rate was calculated as: Mobility (%) = (1 – 48-h scratch distance/initial distance) ×100%.

### Transwell invasion assay

Cells were collected following transfection. A Transwell invasion chamber (Beyotime Institute of Biotechnology; polycarbonate pore membrane with pore size 8 *µ*m) was placed in a 24-well cell culture plate. Matrigel (15 *µ*g/ml; Beyotime Institute of Biotechnology) was placed on the surface of the filter membrane of the Transwell chamber, and following coagulation, RPMI 1640 serum-free medium (Beyotime Institute of Biotechnology) (37°C) was used for hydration for 30 min. 0.25%trypsin (Beyotime Institute of Biotechnology) was used for digestion of cells in the logarithmic growth phase. Following suspension and dilution of serum-free medium, 1×10^5^ cells were inoculated in each chamber containing a volume of 200 *µ*l, and 600 *µ*l complete medium was added to the lower chamber. Following 24 h of incubation, the small chamber was removed. A cotton swab was used to wipe the non-invaded cells from the surface of the microporous membrane. The filter membrane was then fixed with methanol for 20 min. Crystal violet (Shanghai Qiaoxing Trading Corporation, Shanghai, China) staining was performed for 10 min, and under an optical microscope (magnification, x200), eight fields of view were randomly selected to perform the averaged cell count. The above experiment was repeated three times, with three wells per group.

### Statistical analysis

Data were processed with SPSS 17.0 statistical software (SPSS, Inc., Chicago, IL, USA). Continuous values are expressed as the mean ± standard deviation (x±s), and differences between two groups were tested using a small sample *t* test, P<0.05 was considered to indicate a statistically significant difference between values.

## Results

### PCR detection of miR-21 and SPRY2 gene expression in MM cell lines

**Figure 1:**
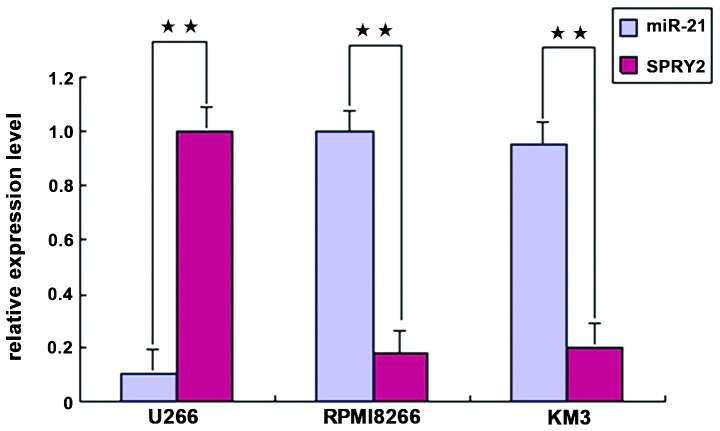
miR-21 and SPRY2 gene expression in MM cell lines. ^**^P<0.01, compared with miR-21. miR, microRNA; SPRY2, sprouty homolog 2.

In the MM cell lines RPMI8226 and KM3, miR-21 expression was high and SPRY2 expression was low. In the U266 cell line, miR-21 expression was low, and SPRY2 expression was high; the differences were statistically significant (P<0.01) (Fig. 1).

### Western blot analysis of miR-21 and SPRY2 protein expression in MM cells

**Figure 2:**
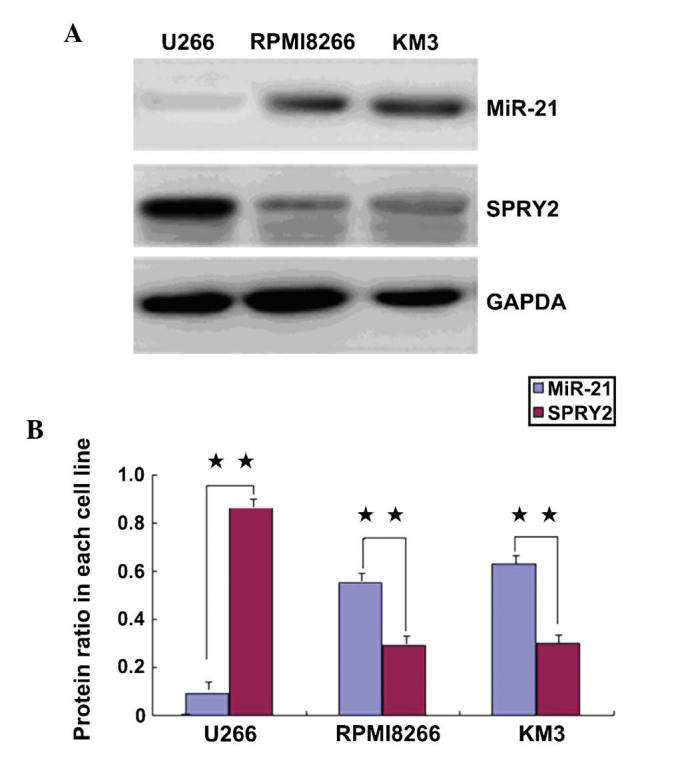
Western blot analysis of miR-21 and SPRY2 protein expression and in MM cell lines. (A) SPRY2 and miR-21 expression levels in the indicated cell lines; (B) Quantified SPRY2 and miR-21 protein levels in each cell line. ^**^P<0.01, compared with miR-21. miR, microRNA; SPRY2, sprouty homolog 2.

Western blot analysis showed that endogenous miR-21 expression in the MM cell lines RPMI8226 and KM3 was high, while SPRY2 expression was significantly lower. Conversely, in the U266 cell line, endogenous miR-21 expression was low and SPRY2 expression was significantly higher (Fig. 2A). The gray values of miR-21 and SPRY2 protein in the respective cell lines showed statistically significant differences (P<0.01) (Fig. 2B).

### Inhibition of miR-21 expression following infection with LV-anti-miR21 in U266 cells

**Figure 3:**
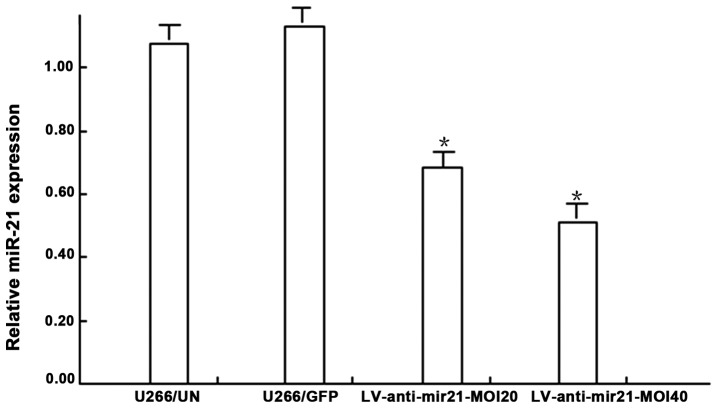
miR-21 expression levels in U266 cells after transfection with miRs. *P<0.05, compared with the U266/un group. miR, microRNA; SPRY, sprouty homolog; UN, untransfected; GFP, green fluorescent protein; LV, lentiviral vector; MOI, multiplicity of infection.

RT-qPCR showed that following transfection of U266 cells, expression levels were as follows: miR-21 expression in the U266/GFP group was higher than that in the U266/un group (P>0.05); miR-21 expression in the U266/LV-anti-miR-21 lentiviral MOI 20 group and the MOI 40 group was significantly lower than that in the U266/un group (P<0.05). miR-21 expression in the U266/LV-anti-miR-21 lentivirus MOI 20 group was higher than that in the MOI 40 group (P>0.05) (Fig. 3).

### SPRY2 expression in MM cells following transfection

**Figure 4:**
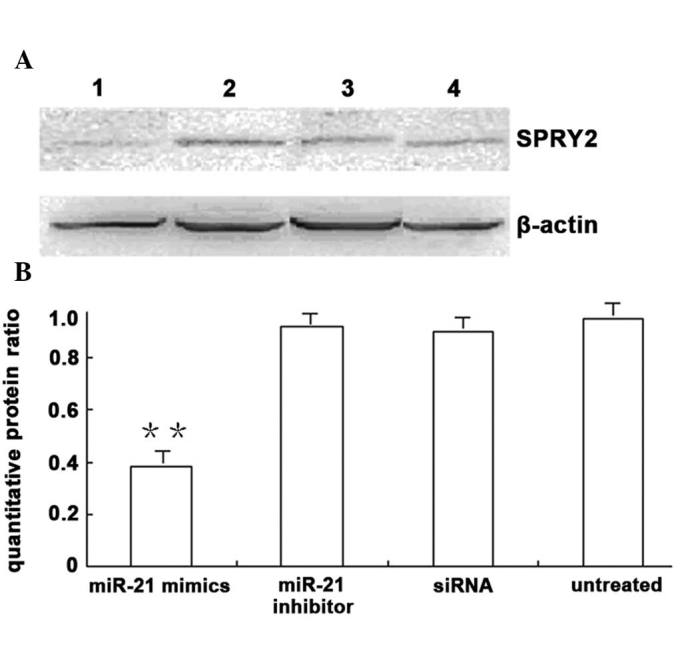
SPRY2 protein expression following transfection of U266 cells with miR-21. (A) SPRY2 protein expression levels in transfected U266 cells in each group. Lanes: 1, miR-21 mimics; 2. miR-21 inhibitor; 3, siRNA; 4, untreated. (B) Quantified protein expression. ^**^P<0.01, compared with the siRNA and untreated groups. miR, microRNA; SPRY2, sprouty homolog 2; siRNA, small interfering RNA.

Western blot analysis showed that SPRY2 protein expression in U266 cells in the transfected miR-21 mimics group was significantly lower than that in the untreated group and the siRNA negative control group (P<0.01) (Fig. 4).

### U266 cells overexpressing reduced SPRY2 proliferative capacity

**Figure 5:**
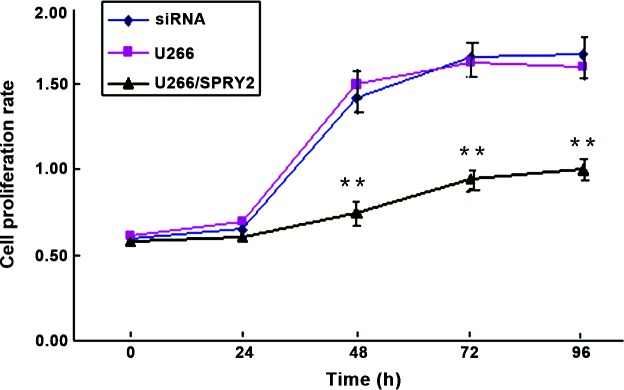
SPRY2 overexpression reduces the proliferation of U266 cells. ^**^P<0.01, compared with the siRNA and U266 groups. SPRY2, sprouty homolog 2; siRNA, small interfering RNA.

The non-transfected group, the negative control group and the SPRY2 plasmid-transfected U266 cells were cultured for four days. An MTT assay showed that the growth of SPRY2 plasmid-transfected U266 cells was significantly decreased at 48, 72 and 96 h, and the proliferation rate decreased significantly (P<0.01). No significant difference in cell growth was noted between the untransfected group and the negative control group (P>0.05) (Fig. 5).

### Effect of miR-21 gene expression on apoptosis of MM

**Figure 6:**
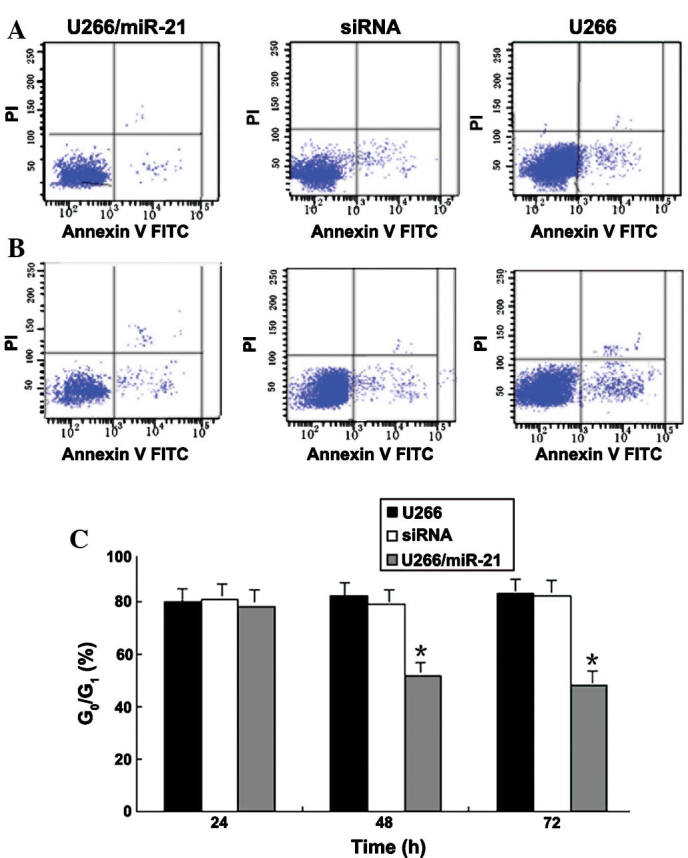
Assessment of U266 cell apoptosis by flow cytometry. (A and B) Dot plots of PI/Annexin V-FITC double stained cells at (A) 48 and (B) 72 h after transfection, showing apoptotic cells in the bottom right window. (C) Cells in G_0_/G_1_ phase in each group. *P<0.05, compared with the siRNA and U266 groups. PI, propidium iodide; FITC, fluorescein isothiocyanate; siRNA, small interfering RNA; miRNA, microRNA.

Flow cytometry showed that 48 and 72 h following transfection of U266 cells with miR-21 mimics, the apoptotic rates were (24.7±1.97 and 38.6±1.56%, respectively) in the U266 group, (27.3±1.72 and 37.3±1.59%, respectively) in the siRNA group and (12.7±1.27 and 22.1±1.63%, respectively) in the U266/miR-21 group. Compared with the two control groups, the apoptotic rate in the U266/miR-21 group was significantly lower, and the cell population in G_0_/G_1_ phase was significantly reduced (P<0.05) (Fig. 6).

### Effects of miR-21 gene expression on migration of MM cells

**Figure 7:**
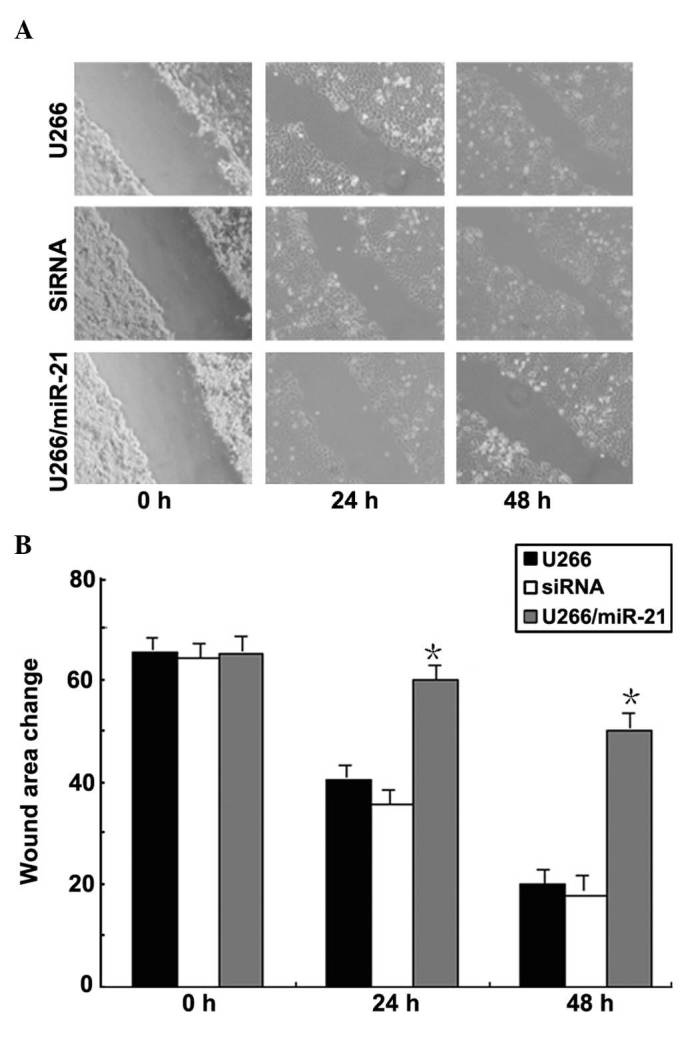
Wound healing assay using the U266 cell line. (A) Cell migration ability in scratch damage experiments of each group (scale, 500 *µ*m; magnification x4). (B) Quantification of wound area changes 0, 24 and 48 h after scratch damage. ^*^P<0.05, compared with the siRNA and U266 groups. siRNA, small interfering RNA; miR, microRNA.

Scratch test results showed that the edge of the wound was neat subsequently following scratching. Following 24 and 48 h of incubation, the cell processes significantly increased and migrated to the damaged area, whose size gradually decreased. The ability of transfected cells to migrate into the wound area significantly decreased when compared with that of the non-transfected and negative control groups (P<0.05) (Fig. 7).

### Effects of miR-21 gene expression on the invasion of the MM cells

**Figure 8:**
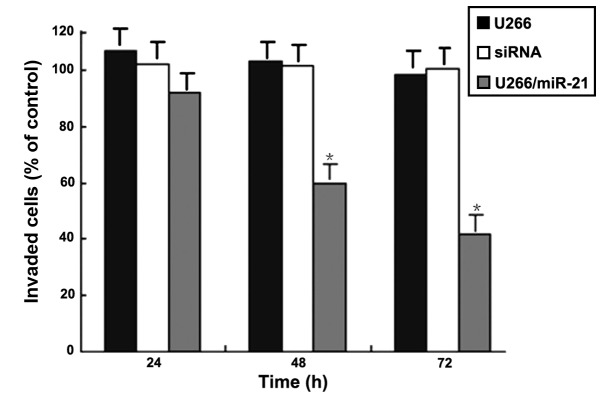
U266 cell invasion following transfection with miR-21 mimics. ^*^P<0.05, compared with siRNA and U266 groups. siRNA, small interfering RNA; miR, microRNA.

The Transwell invasion assay showed changes in U266 cell invasion at 24, 48 and 72 h following transfection with miR-21 mimics. At the same time-point, the differences in numbers of U266 cells which transgressed through the Matrigel-covered polyphosphate membrane were not statistically significant between the two control groups (P>0.05). At 48 and 72 h, the number of U266 cells which passed through the Matrigel-covered polyphosphate membrane in the transfected group significantly decreased as compared with that in the non-transfected and negative control groups (P<0.05) (Fig. 8).

## Discussion

An ideal tumor marker should be easily detectable using non-invasive methods. The use of miR-21 as a molecular marker has been the focus of numerous studies (11–13). It is abnormally expressed in a variety of malignant tumors and has a pivotal regulatory role in the development of tumors (12–14). Studies showed that the overexpression of miR-21 was associated with proliferation, metastasis and prognosis of MM (15), non-Hodgkin’s lymphoma, leukemia (16) and various non-hematologic solid tumors (17). miR-21 was shown to be able to regulate SPRY2 expression (5), and the structure of SPRY proteins was shown to be rich in C-terminal of cysteine, highly evolutionarily conserved (6), and its target region was positioned in the activated cell membrane, with a strong variation in the N-terminal region (7). Several studies showed that SPRY2 gene expression was downregulated and inhibited in prostate cancer, breast cancer, malignant glioma and other tumor types, leading to uncontrolled and overactivated mitogen-activated protein kinase/extracellular-regulated kinase (MAPK/ERK) signaling in tumor cells (7,18–20). Therefore, SPRY2 was considered an oncogene involved in MAPK/ERK signaling. In the present study, RT-qPCR and western blot analyses showed that in the MM cell lines with high endogenous miR-21 expression (RPMI8226 and KM3), SPRY2 expression was low. Conversely, in the U266 cell line exhibiting low endogenous miR-21 expression, SPRY2 expression was higher. The gray values of miR-21 and SPRY2 protein in the respectve cell lines showed significant differences (P<0.01). This illustrated that miR-21 may be negatively correlated with SPRY2 in MM cells. To the best of our knowledge, the effects of miR-21-mediated downregulation of SPRY2 gene expression on the proliferation and invasion of MM cells as well as the underlying molecular mechanism have not been previously reported. For this reason, in the present study, the LV-anti-miR-21 vector was constructed, and MM cell lines rediced expression levels of SPRY2 were successfully established. In LV-anti-miR-21-infected U266 cells, miR-21 expression was significantly inhibited (P<0.05) and SPRY2 protein expression was significantly increased (P<0.01). This further confirmed that miR-21 is able to regulate the expression of SPRY2.

The results indicated that high levels of miR-21 and downregulation of SPRY2 may inhibit cell proliferation, migration and invasion, and promote apoptosis. It can be concluded that miR-21 is able to downregulate SPRY2 expression; it is therefore indicated that in the development of MM, low miR-21 levels lead to the promotion of cell proliferation, invasion and metastasis, and the inhibition of apoptosis. miR-21 is therefore a potential biological target, which may be upregulated to increase apoptosis signaling, and therefore may be used to treat and prevent the generation of tumors.

The main pathogenesis of cancer involves deregulation of the cell cycle, leading to unlimited regulation of cell growth. Apoptosis is induced following insults originating from intracellular processes of the external environment. It is the active cell suicide process controlled by apoptotic proteins, which participate in various parallel signaling pathways and/or activation cascades (21). A previous study showed that SPRY2 inhibits MAPK/ERK activation as well as interleukin-6-stimulated MM cell growth (22). The ability of SPRY2 to inhibit MAPK/ERK signaling pathway activation suggested that SPRY2 functions as a tumor suppressor gene in MM cells. The MTT assay in the present study showed that SPRY2 overexpression decreased the proliferation of U266 cells (P<0.01). Flow cytometric analysis showed that 48 and 72 h after transfection, the apoptotic rate in the U266/miR-21 group was significantly decreased, and the G_0_/G_1_-phase population was significantly reduced, suggesting that transfected miR-21 mimics can enhance the tolerance of U266 cells to apoptosis. These results indicated that miR-21 downregulated SPRY2 gene expression and promoted the proliferation and migration functions of MM cells *in vitro*. These results provided a molecular mechanisms underlying the occurrence and development of MM.

The molecular mechanisms of invasion and metastasis, which are malignant processes in cancer tissues, are complex multi-step processes. The occurrence and development of metastases is based on complex interactions between tumor cells and the host, and the underlying mechanisms have remained to be fully elucidated, as a large number of genes and proteins are involved (23–25). Exploring the mechanism of invasion and metastasis of MM at the molecular level, to determine the prognosis and prolonged survival time as well as to improve the survival rate has been the focus of studies on MM. In the present study, a wound healing assay showed that the migration ability of the cells significantly decreased following miR-21 transfection/SPRY2 downregulation (P<0.05). A Transwell invasion assay demonstrated that the number of U266 cells which transgressed through a Matrigel-covered polyphosphate membrane significantly decreased. These results indicated that the miR-21-mediated downregulation of SPRY2 expression inhibited the migration and invasion of MM cells and may therefore have a beneficial effect on MM. Further studies are required, using *in vivo* experiments including tumor occurrence in nude mice and transfection by intravenous injection. The present study provided an experimental basis for further mechanistic studies on MM cell migration and invasion.

In conclusion, RNA interference technology was used to establish a stably SPRY2-silenced MM cell line. The results suggested that miR-21 downregulated SPRY2 gene expression and decreased cell proliferation and invasion of MM cells *in vitro*. The present study provided experimental evidence and a theoretical basis for the development of clinical treatments of MM, and its prospects and potential clinical application deserve further study.
